# Quantifying Signal Quality From Unimodal and Multimodal Sources: Application to EEG With Ocular and Motion Artifacts

**DOI:** 10.3389/fnins.2021.566004

**Published:** 2021-02-12

**Authors:** David O. Nahmias, Kimberly L. Kontson

**Affiliations:** ^1^Office of Science and Engineering Laboratories, Division of Biomedical Physics, Center for Devices and Radiological Health, U.S. Food and Drug Administration, Silver Spring, MD, United States; ^2^Department of Electrical and Computer Engineering, University of Maryland, College Park, MD, United States

**Keywords:** signal quality, artifact detection, electroencephalography, quantitative EEG, machine learning

## Abstract

With prevalence of electrophysiological data collected outside of the laboratory from portable, non-invasive modalities growing at a rapid rate, the quality of these recorded data, if not adequate, could affect the effectiveness of medical devices that depend of them. In this work, we propose novel methods to evaluate electrophysiological signal quality to determine how much of the data represents the physiological source of interest. Data driven models are investigated through Bayesian decision and deep learning-based methods to score unimodal (signal and noise recorded on same device) and multimodal (signal and noise each recorded from different devices) data, respectively. We validate these methods and models on three electroencephalography (EEG) data sets (*N* = 60 subjects) to score EEG quality based on the presence of ocular artifacts with our unimodal method and motion artifacts with our multimodal method. Further, we apply our unimodal source method to compare the performance of two different artifact removal algorithms. Our results show we are able to effectively score EEG data using both methods and apply our method to evaluate the performance of other artifact removal algorithms that target ocular artifacts. Methods developed and validated here can be used to assess data quality and evaluate the effectiveness of certain noise-reduction algorithms.

## 1. Introduction

Advancements in and availability of wearable technologies that can readily collect electrophysiological data from individuals in both controlled laboratory and real-world settings have been growing rapidly. As such, both the volume of available biometric data and its potential utility, if properly understood, are also increasing. If these data are to be effectively applied and correctly interpreted, it is important to understand the quality of data being recorded. In this context, quality is defined by how much of the acquired signal is from the source of interest and not noise from external or internal (i.e., other physiological) sources. Unlike in clinical or research settings, electrophysiological data collected in the real world is often contaminated with noise that does not represent the physiological signal of interest.

In the case of electrocardiography (ECG), an example of an electrophysiological signal, recordings often include electromyography (EMG), and movement, among other noise sources. For ECG, there have been several efforts in developing methods to assess signal quality (Satija et al., 2018). However, for another type of electrophysiological signal, electroencephalography (EEG), there has been little research on developing signal quality metrics. EEG, which measures brain electrical signals from the scalp, is a common neuro-monitoring technique used in both clinical and research settings. Depending on the application, it would be beneficial to evaluate the quality of data and know how clean electrophysiological recordings are before attempting to analyze it or use it as input to a model (Lai et al., 2018). For clinical applications where we need these data to be reliable, consistent, and informative, the presence of noise that corrupt the signal of interest can degrade the effectiveness of diagnostic tools and brain-machine interfaces. With EEG, ocular activity [measurable by electrooculography (EOG)], muscle activity (measurable by EMG), cardiac pulses (measurable by ECG), and movement [measurable by inertial measurement units (IMU)] are examples of such noise that can often corrupt the purity of neural activity targeted by EEG recordings (Islam et al., 2016). Creating metrics to determine the quality of non-invasive electrophysiological recordings would inform those using the data how representative it is of the desired physiological source and not riddled with noise from sources not of interest.

There have been a few approaches to scoring EEG signal quality. With the ability to directly acquire signals from noise sources, there have been greater successes in applying artifact removal algorithms when the noise signal is known (Kilicarslan et al., 2016; Kilicarslan and Vidal, 2019). In many situations, however, it may not be possible to directly measure the source of artifacts, making the process by which those artifacts are removed arduous and more error-prone. To better assess data quality where noise sources are not available, we can leverage data from studies with the appropriate data to generate models that characterize and score electrophysiological recordings. One previous method calculated 11 different features of EEG that were used to identify clean EEG recording segments by thresholding these signal parameters (Daly et al., 2012). Data across four data sets were used to determine these thresholds with *n* = 58. Based on these thresholds, features values from new EEG signals were categorized as either clean or not clean. In another study, three quantitative EEG features were used to assess signal quality to obtain three scores which were combined into one score (Hu et al., 2013). The data used was from the OPTIMI data set with *n* = 90, but the method may need modification to be generalized to other headsets. Recently, machine learning was applied to this problem using 114 features from the EEG (Grosselin et al., 2019). The EEGs were classified using several classification approaches, along with feature selection and a five-fold cross validation into three quality levels: low, medium, and high. This study used EEG across five data sets with *n* = 43. These prior works have generally used a limited number of quantitative features, have not used noise sources directly, and/or have characterized signal quality into no more than three discrete categories.

Based on these gaps, the aims of this work are to (1) develop a continuous scoring method for data from a unimodal source when the noise can be measured directly from the same modality and apply it to EEG with ocular artifacts, (2) develop a continuous scoring method for data when the noise can only be measured from another modality, requiring multimodal sources, and apply it to EEG with motion artifacts, and (3) apply our developed scoring metric to evaluate artifact removal algorithms, specifically comparing two artifact removal algorithms that target ocular artifacts.

This work proposes new methods to create a metric to quantify quality of electrophysiological data. Our first proposed approach is targeted at applications when the noise source can be recorded directly using the same measurement tool, i.e., unimodal data such as EEG and EOG, which are both recorded from electrodes on the head. Our second approach would be needed when there are noise sources that cannot be recorded directly and can only be quantified by other means, i.e., multimodal data such as EEG and motion, which require both EEG electrodes and IMUs. We propose a feature-based Bayesian approach to score EEG with ocular artifacts since EEG and EOG can be directly measured through same set of electrodes. Recently deep learning, specifically deep convolutional neural networks (DCNN), have shown state-of-the-art results and superb effectiveness in EEG applications (Roy et al., 2019). As such, we next present a deep learning-based approach to score EEG with motion artifacts since motion cannot be directly recorded with electrodes but rather is quantified by IMU or other motion tracking tools.

Further, we validate and apply our scoring metric to evaluate the effectiveness of different artifact removal algorithms. We hypothesize that data cleaned with other artifact removal algorithms will obtain higher scores than before they were processed. Methods to compare the performance of EEG artifact removal algorithms have not been well-developed and currently rely on either visual inspection or synthetic data (Islam et al., 2016). Since ocular artifacts are the most common noise targeted by artifact removal algorithms for EEG, we score recorded data with noise present and data after being processed by different ocular artifact removal algorithms (Jiang et al., 2019). These scoring methods could be used to evaluate the effectiveness of noise removal algorithms by comparing scores of EEGs processed by different methods.

## 2. Methods

**Figure 1:**
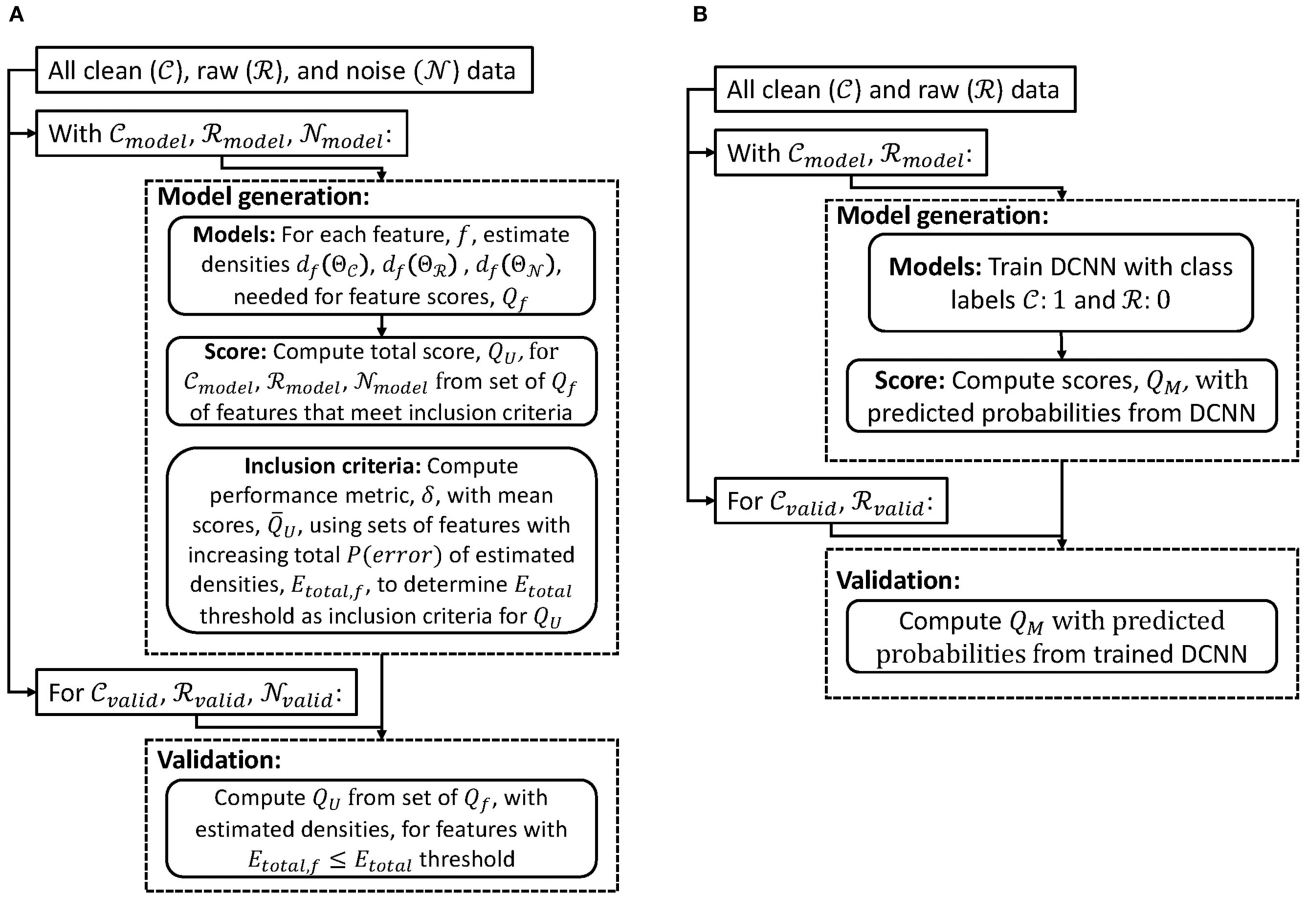
Flowcharts of model generation and validation of scoring methods for data with **(A)** unimodal and **(B)** multimodal sources.

We first introduce a scoring method for cases when the noise source can be recorded directly by the same modality (section 2.1). We next describe a scoring method when the noise source cannot be measured directly through the same recording modality (section 2.2). Both methods are designed to generate a score 0 ≤ *Q* ≤ 1 (*Q*_*U*_ for data with unimodal source and *Q*_*M*_ for data with multimodal sources). A score of zero would imply that the data is entirely noise or from sources not of interest, while a score of one would mean the data is entirely from the desired electrophysiological source. We applied these methods to EEG with different types of noise sources. In general, we used common average re-referencing as a pre-processing step so that models generated could be applied to signals with different recording parameters (e.g., reference or ground channels). Figure 1 shows a high-level processing pipeline of model generation and validation of scoring methods for data with unimodal (Figure 1A) and multimodal (Figure 1B) sources. Finally, we outline two different noise removal algorithms and how our scoring method could be used to evaluate their performance (section 2.3).

### 2.1. Scoring Data With Unimodal Source

In cases where the noise source can be measured directly from the same recording modality (referred to as “unimodal method”), it is possible to compare quantitative features of both the signal of interest and the noise. After computing these features and their corresponding scores, we detail how to identify which features would be most effective to score data quality.

#### 2.1.1. Scoring Method for Data With Unimodal Source

We begin by computing several quantitative features (30 initial features were used in this study) for data without noise, hereafter referred to as clean data (C), raw recorded data with noise present, referred to as raw data (R), and the noise source, referred to as noise data (N).

With the collection of quantitative features for each recording, we fit a distribution for each feature for each type of data, (clean, raw, and noise) through kernel density estimations (KDE). Our KDE based models are computed using Gaussian kernels and Scott's rule for bandwidth size. For each source of data and feature, *f*, a set of parameters, Θ, and subsequently distributions *d*_*f*_(Θ_*C*_), *d*_*f*_(Θ_*R*_), and *d*_*f*_(Θ_*N*_), are estimated.

To obtain a sub-score from each feature, we use the Bayesian decision critical value, $v_{f}^{*}$, which minimizes the probability of error between each set of estimated distributions, $d_{f}\left(\Theta_{C}\right)$ and $d_{f}\left(\Theta_{N}\right)$ (Duda et al., 2000). For each feature used, if the mean value of N is less than the mean value of C, a sub-score using that recording's feature's value, *v*_*f*_, is obtained by

(1) $$\begin{array}{l} Q_{f}\left(v_{f}\right)=\{\begin{array}{l} \frac{1}{2}+\frac{1}{2}P\left(v_{f}>x_{i}\;|\;x_{i}\in C\right),\text{if }v_{f}\geq v_{f}^{*}\\ \frac{1}{2}P\left(v_{f}\leq x_{i}\;|\;x_{i}\in N\right),\text{if }v_{f}<v_{f}^{*} \end{array} \end{array}$$

In essence, if $v_{f}\geq v_{f}^{*}$ then *Q*_*f*_(*v*_*f*_) represents the proportion of values in C less than *v*_*f*_ scaled between [0.5, 1], or if $v_{f}<v_{f}^{*}$ then *Q*_*f*_(*v*_*f*_) represents the proportion of values in N greater than *v*_*f*_ scaled between [0, 0.5]. If the mean value of features in N is greater than the mean value of features in C, then the inequalities in Equation (1) are reversed appropriately. To obtain the final quality score for our unimodal method a set of features for a recording, *V*,

(2) $$\begin{array}{l} Q_{U}\left(V\right)=\frac{1}{F}\overset{F}{\underset{f=1}{\sum }}Q_{f}\left(v_{f}\right),\\ \;v_{f}\in V\text{ for features }f\text{ that meet inclusion criteria}, \end{array}$$

where *F* is the number of computed features used to obtain the overall score.

We next develop a method to identify an inclusion criterion for which features would be best for determining *Q*_*U*_. We compute three probabilities of errors for each feature for each set of data as follows:

(3) $$\begin{array}{l} P\left(error|C,f\right)=P\left(d_{f}\left(x_{i}|\Theta_{C}\right)<d_{f}\left(x_{i}|\Theta_{N}\right)\;|\;x_{i}\in C\right) \end{array}$$

(4) $$\begin{array}{l} P\left(error|R,f\right)=P\left(d_{f}\left(x_{i}|\Theta_{C}\right)<d_{f}\left(x_{i}|\Theta_{N}\right)\;|\;x_{i}\in R\right)\\ \;=1-P\left(d_{f}\left(x_{i}|\Theta_{N}\right)\leq d_{f}\left(x_{i}|\Theta_{C}\right)\;|\;x_{i}\in R\right) \end{array}$$

(5) $$\begin{array}{l} P\left(error|N,f\right)=P\left(d_{f}\left(x_{i}|\Theta_{N}\right)<d_{f}\left(x_{i}|\Theta_{C}\right)\;|\;x_{i}\in N\right) \end{array}$$

Ideally, the clean and noise distributions should be completely separable, with a probability of error of zero, while the distribution of raw data should be a combination of values from the clean and noise distribution and thus should have a probability of error of 0.5 between both the clean and noise distributions. Thus, we can evaluate the utility of each feature by computing the error of each estimated distribution from the ideal error, referred to as total error. We define the total error for each feature as

(6) $$\begin{array}{l} \;E_{total,f}\;=P\left(error|C,f\right)+|0.5-P\left(error|R,f\right)|+P\left(error|N,f\right) \end{array}$$

where 0 ≤ *E*_*total, f*_ ≤ 2.5.

Lower *E*_*total, f*_ represent features best suited for scoring signal quality and only features with low enough *E*_*total, f*_ should be used. Finally, we formulate a metric to determine the inclusion criteria of how low *E*_*total, f*_ of all features should be, *E*_*total*_ threshold. We define a measure of the error from the ideal solution,

(7) $$\begin{array}{l} \delta =|0.75-{\bar{Q}}_{U}\left(C\right)|+|0.50-{\bar{Q}}_{U}\left(R\right)|+|0.25-{\bar{Q}}_{U}\left(N\right)| \end{array}$$

where ${\bar{Q}}_{U}$ is the mean *Q*_*U*_(*V*) across all data of each type, $V_{C}\in C,V_{R}\in R,\text{and}V_{N}\in N$, features from clean data, raw data, and noise data, respectively.

To interpret *Q*_*U*_ from this method effectively we wish to have the mean of $Q_{U}\left(V_{C}\right)$, ${\bar{Q}}_{U}\left(C\right)$, be 0.75 and have range between 0.5 and 1, the mean of $Q_{U}\left(V_{R}\right)$, ${\bar{Q}}_{U}\left(R\right)$, to be 0.50 and have range between 0.25 and 0.75, and the mean of $Q_{U}\left(V_{N}\right)$, ${\bar{Q}}_{U}\left(N\right)$, to be 0.25 and have range between 0 and 0.50. These constraints and parameters make it such that ideally there will be no overlap between C and N while R will overlap approximately half with C and half with N.

Therefore, to obtain an optimal *E*_*total*_ threshold, we calculate δ with increasing values of *E*_*total*_ thresholds to observe when δ begins to increase. From this analysis, we only use features with *E*_*total, f*_ lower than the determined threshold value as an inclusion criteria for Equation (2).

#### 2.1.2. Data With Unimodal Source

As the unimodal approach is data-driven, we present here data used to generate scoring parameters and subsequently validate this method. We focus on noise from eye-movement, EOG, since for high density EEGs they are generally captured directly by electrodes placed near the eyes.

The data set used in this study was obtained from the University of Houston and contained EEG and EOG recordings (sampled at 100 Hz) as well as motion capture from eleven subjects walking on a treadmill for 6 min (Kilicarslan and Vidal, 2019). Of these eleven subjects, eight were used for this unimodal approach because of the availability and consistency of data. EEG were recorded with a 58 electrode array following labels from the extended 10-20 system while EOG were recorded with four electrodes placed above and below each eye.

A robust noise removal method developed by the University of Houston research group directly used available noise sources to remove them from the recorded EEG (Kilicarslan et al., 2016). This noise removal algorithm targeting EOG noise used an *H*^∞^ filtering formulation since it guarantees robustness where small modeling errors and external noise do not cause large estimation errors (Hassibi and Kailath, 1995). This algorithm and subsequent study used four EOG channels directly recorded as reference disturbance input. The strength and effectiveness of this algorithm are shown to out-perform other common ocular artifact removal techniques (Kilicarslan and Vidal, 2019). For more detailed derivation of the *H*^∞^ filtering formulation and algorithm targeting EOG noise used in this study, the following reference can be reviewed (Kilicarslan et al., 2016).

The *H*^∞^ EOG cleaning algorithm was applied to the 6 min of recording for the eight subjects. We then separated data from EEG channels and EOG channels. This gave us 58 channels with 6 min of recording of both clean EEG data and raw EEG, as well as four channels with 6 min of EOG data. For EEG, we used 30 features on segmented data of 1-min epochs since these features have been shown to be stable with these higher epoch lengths in previous quantitative EEG studies (Nahmias et al., 2019). This yielded $C_{Eye}$ and $R_{Eye}$ with *n* = 2, 784 (58 channels ×6 min ×8 subjects = 2,784), and $N_{Eye}$ with *n* = 192 (4 channels ×6 min ×8 subjects = 192), where each sample was of size 30 ×1 (30 features), for our method scoring data with unimodal source.

#### 2.1.3. Model Generation for Data With Unimodal Source: EEG With Ocular Artifacts

To obtain scoring models for this data we separated 90% of the data randomly to generate the models and reserved 10% of the data to test and validate the results. We present 10-fold cross-validated values from ten generated models from different samplings of 90% of the data, denoted with the subscript “*model,”* that resulted in $C_{Eye,model}$ and $R_{Eye,model}$, both with *n* = 2506, and $N_{Eye,model}$, with *n* = 173. We report the mean results as well as the mean standard deviation across samples for the 10-fold cross-validation.

As mentioned, we computed 30 features for both EEG and EOG data. For spectral features, the Fourier transforms were taken on the pre-processed recording after which various spectral features were computed. The power spectral density (PSD) of frequency bands commonly analyzed in the EEG were estimated using the periodogram. The ranges of the frequency bands applied in this study were as follows: δ(delta):1−4 Hz, θ(theta):4−8 Hz, α(alpha):8−12 Hz, μ(mu):12−16 Hz, β(beta):16−25 Hz, γ(gamma):25−40 Hz (Schomer and Lopes da Silva, 2010). Both absolute powers and relative powers were computed, with relative power equal to the power in a frequency band divided by the total power. The entropy of the periodogram, and entropy of the normalized periodogram, were found using the Shanon entropy definition (Blanco et al., 2014). In addition to the spectral features, the following time domain features, directly from the pre-processed EEG signal, were computed: entropy of the normalized signal, mean thresholded Lempel-Ziv complexity (LZC), minimum value, maximum value, median, mean, variance, standard deviation, skew, kurtosis, curve length, energy, non-linear energy, sixth power, sum, mobility, complexity.

**Figure 2:**
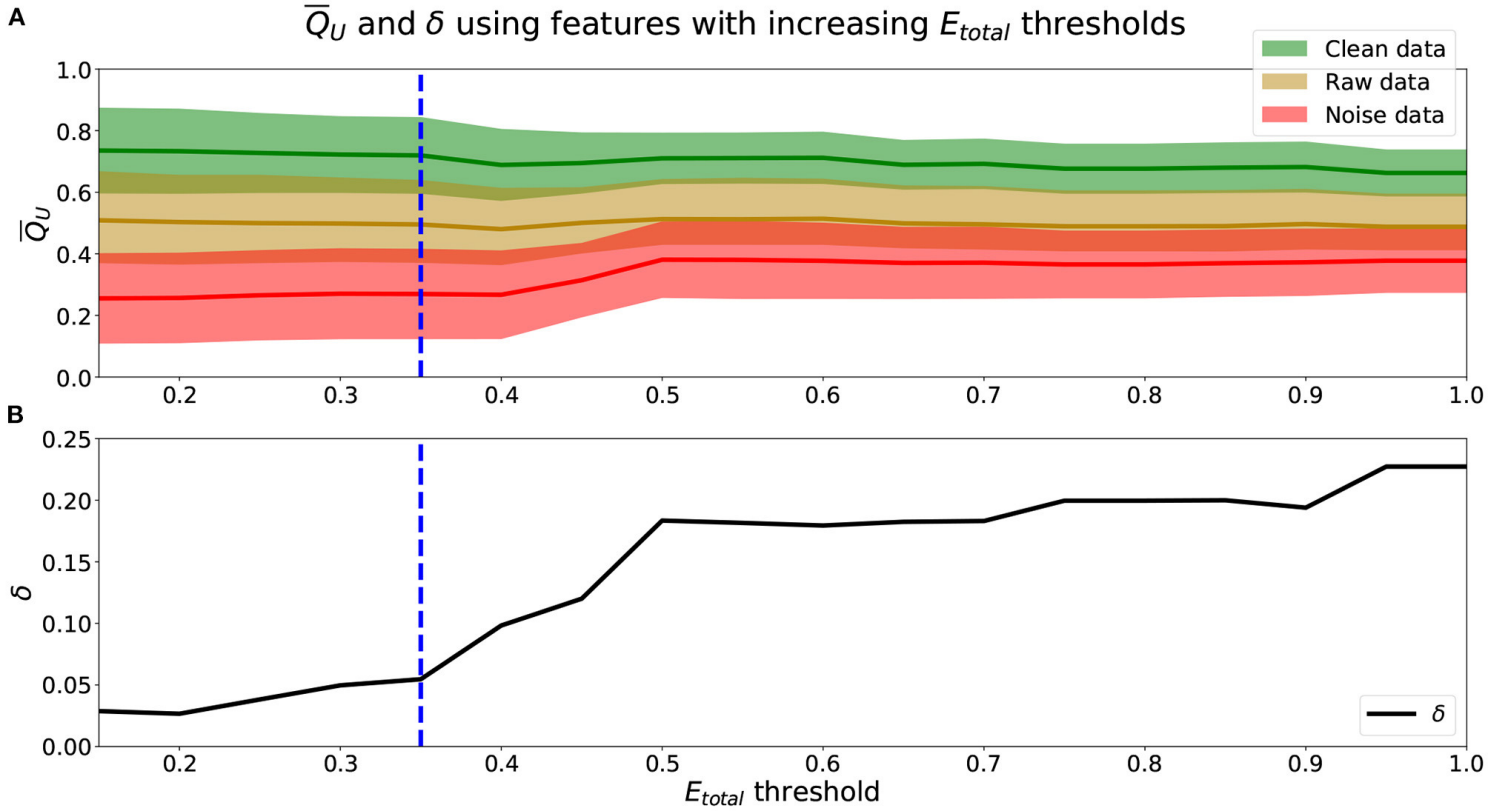
**(A)** ${\bar{Q}}_{U}$ means (lines) and standard deviations (shaded area) using features with increasing *E*_*total*_ thresholds. Green represents ${\bar{Q}}_{U}\left(C_{Eye,model}\right)$, yellow ${\bar{Q}}_{U}\left(R_{Eye,model}\right)$, and red ${\bar{Q}}_{U}\left(N_{Eye,model}\right)$. **(B)** δ values using ${\bar{Q}}_{U}$ obtained from increasing *E*_*total*_ thresholds. **(A,B)** Blue dashed-line marks a threshold of *E*_*total*_ = 0.35, where δ begins to increase more rapidly.

To identify the appropriate *E*_*total*_ threshold for the set of *Q*_*f*_ that will be used to calculate *Q*_*U*_ (Equation 2), we analyze the relationship between the *E*_*total*_ threshold (Equation 6) and ${\bar{Q}}_{U}$ along with their associated δ (Equation 7) in Figure 2.

**Figure 3:**
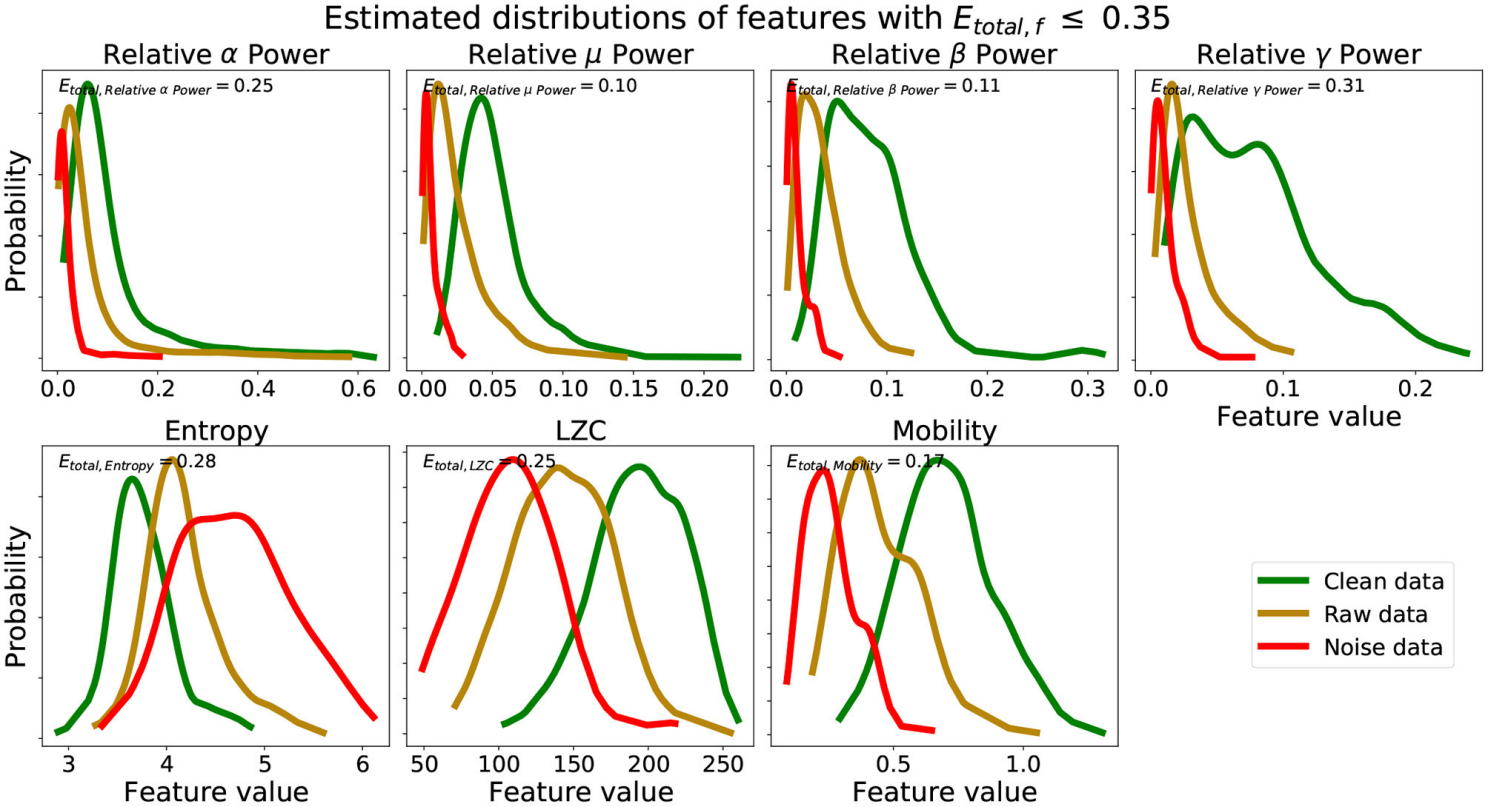
Estimated distributions clean, raw, and noise data for features with *E*_*total, f*_ ≤ 0.35. *E*_*total, f*_ for set of estimated distributions of each feature are also shown.

We see from Figure 2A that using scores from features with lower *E*_*total, f*_ yielded better performing mean scores across data, ${\bar{Q}}_{U}$. Scores of $N_{Eye,model}$ were lower and closer to 0.25, $R_{Eye,model}$ were closer to 0.50, and $C_{Eye,model}$ were higher and closer to 0.75. Further, from Figure 2B, the lower the *E*_*total*_ threshold applied, the lower the corresponding δ value. The best performing and most informative ${\bar{Q}}_{U}$ values were obtained with an inclusion criteria of either a threshold of *E*_*total*_ ≤ 0.20 or *E*_*total*_ ≤ 0.35. Table 1 shows our probabilities of errors (Equations 3–5) and *E*_*total, f*_ (Equation 6) for each of the 30 features, where features with *E*_*total, f*_ ≤ 0.35 are shaded and in bold. Further, to show how these features were distributed and data types appropriately mixed and separated, we show in Figure 3, the estimated distributions of $C_{Eye,model}$, $R_{Eye,model}$, and $N_{Eye,model}$ for features with *E*_*total, f*_ ≤ 0.35.

**Table 1:** Probabilities of error (*P*(*error*)) and total error for estimated densities of each feature (*E*_*total, f*_) from $C_{Eye,model}$, $R_{Eye,model}$, and $N_{Eye,model}$ used in unimodal method.

	**qEEG features**	**P(error|C,f)**	**P(error|R,f)**	**P(error|N,f)**	***E*_*total, f*_**
Spectral	Relative δ Power	0.12	0.18	0.73	1.17
	Relative θ Power	0.14	0.50	0.33	0.47
	**Relative α Power**	**0.11**	**0.55**	**0.09**	**0.25**
	**Relative μ Power**	**0.04**	**0.54**	**0.02**	**0.10**
	**Relative β Power**	**0.06**	**0.54**	**0.02**	**0.11**
	**Relative γ Power**	**0.15**	**0.60**	**0.05**	**0.31**
	Absolute δ Power	0.02	0.17	0.36	0.71
	Absolute θ Power	0.06	0.13	0.43	0.86
	Absolute α Power	0.23	0.23	0.64	1.14
	Absolute μ Power	0.23	0.25	0.72	1.20
	Absolute β Power	0.62	0.68	0.24	1.04
	Absolute γ Power	0.84	0.87	0.10	1.31
	Spectral Entropy	0.17	0.63	0.06	0.36
I.T.	**Entropy**	**0.14**	**0.51**	**0.13**	**0.28**
	**LZC**	**0.10**	**0.59**	**0.06**	**0.25**
Statistical	Minimum	0.07	0.37	0.32	0.52
	Maximum	0.10	0.47	0.31	0.44
	Median	0.00	0.86	0.12	0.48
	Mean	0.00	0.87	0.09	0.46
	Variance	0.07	0.26	0.26	0.57
	SD	0.11	0.41	0.17	0.37
	Skew	0.15	0.42	0.39	0.63
	Kurtosis	0.11	0.21	0.51	0.91
Signal Shape	Curve Length	0.29	0.27	0.61	1.13
	Energy	0.00	0.31	0.47	0.66
	Non-linear Energy	0.19	0.18	0.65	1.15
	Sixth Power	0.00	0.27	0.59	0.83
	Sum	0.00	0.87	0.09	0.46
	**Mobility**	**0.07**	**0.56**	**0.04**	**0.17**
	Complexity	0.27	0.47	0.40	0.70

*Features with E_total, f_ ≤ 0.35 in bold. I.T., Information theoretic; LZC, Lempel-Ziv complexity; SD, Standard deviation*.

We see from Table 1 that the three features with the lowest *E*_*total, f*_ were relative μ power, relative β power, and mobility, followed by relative α power, relative γ power, entropy, and LZC. Visually, we see that features with *E*_*total, f*_ ≤ 0.35 show the following similar traits (Figure 3): (1) the clean data and noise data distributions had little overlap, (2) the mean value of the raw data distributions were close to the critical value (Equation 1), and (3) all data distributions were generally smooth and had a single mode. We verify numerically in Table 2 the appropriate *E*_*total*_ inclusion criteria threshold by using sets of features with increasing *E*_*total*_ thresholds.

**Table 2:** Mean scores ${\bar{Q}}_{U}$ ± standard deviation and δ values of unimodal method across model generation cross-validation using features with incremental *E*_*total*_ thresholds.

**Data**	**${\bar{Q}}_{U}$**	**${\bar{Q}}_{U}$**	**${\bar{Q}}_{U}$**
	**(*E*_*total, f*_ ≤ 0.20)**	**(*E*_*total, f*_ ≤ 0.35)**	**(*E*_*total, f*_ ≤ 0.50)**
$C_{Eye,model}$	0.73 ± 0.14	**0.72 ± 0.13**	0.71 ± 0.08
$R_{Eye,model}$	0.50 ± 0.15	**0.49 ± 0.14**	0.51 ± 0.13
$N_{Eye,model}$	0.26 ± 0.15	**0.27 ± 0.15**	0.38 ± 0.12
δ	0.03	**0.06**	0.18

*Values with chosen threshold, E_total, f_ ≤ 0.35, in bold*.

**Figure 4:**
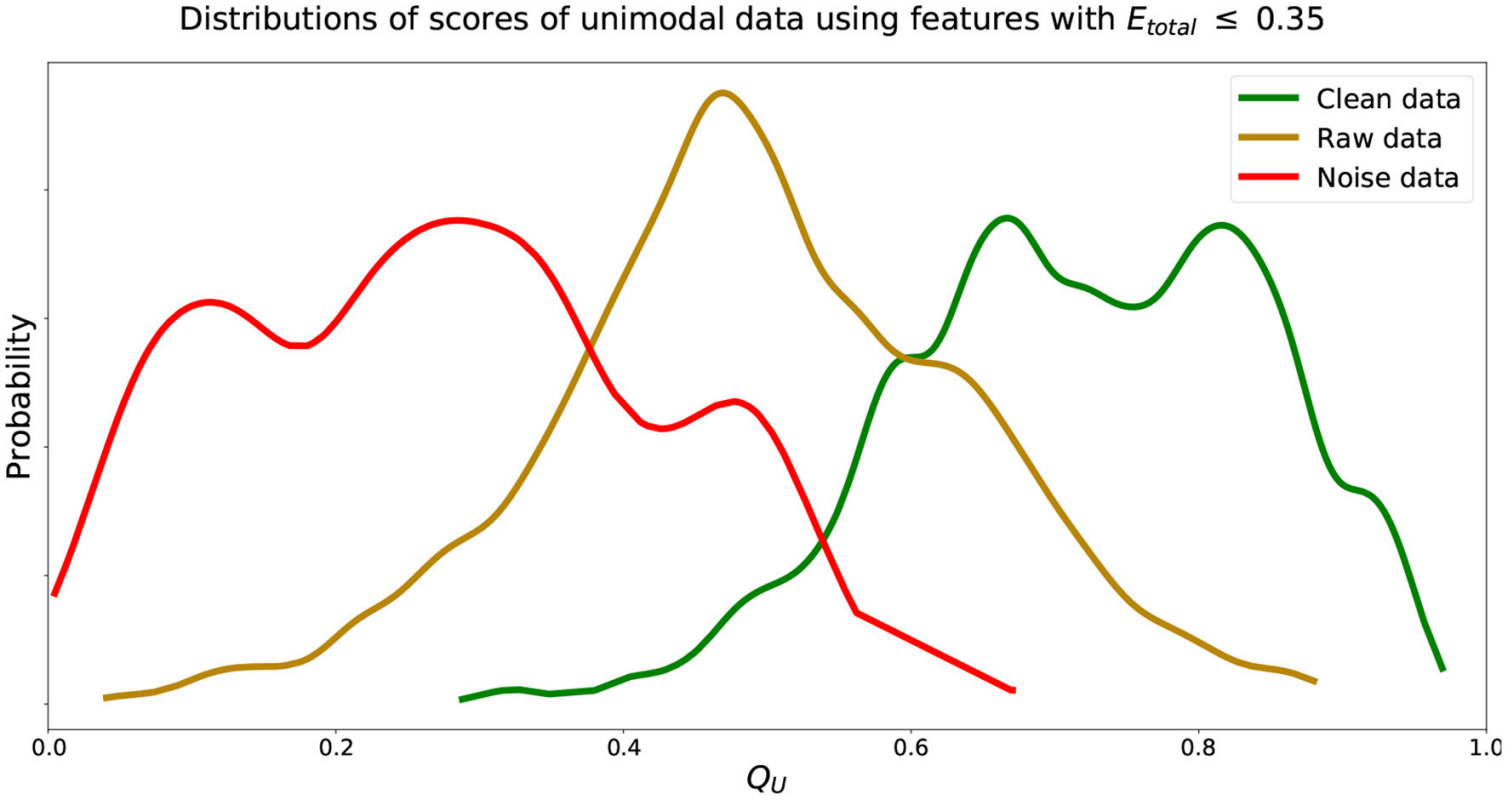
Estimated distributions of *Q*_*U*_ for $C_{Eye,model}$, $R_{Eye,model}$, and $N_{Eye,model}$ using features with *E*_*total, f*_ ≤ 0.35. Distributions of *Q*_*U*_ of $C_{Eye,model}$ and $N_{Eye,model}$ intersect at *Q*_*U*_ = 0.53. Distribution of *Q*_*U*_ of $R_{Eye,model}$ centered at 0.47.

We find that increasing the *E*_*total*_ threshold from 0.20 to 0.35 and including features with 0.20 < *E*_*total, f*_ ≤ 0.35 did not have much of an impact in performance since δ only increased by 0.03. However, further including features with 0.35 < *E*_*total, f*_ ≤ 0.50 did seem to affect score more negatively since δ further increased by 0.12. Thus, to include more features and capture more characteristics of the signals we set the inclusion criteria of features' *Q*_*f*_ to use when computing *Q*_*U*_ (Equation 2) to features with *E*_*total, f*_ ≤ 0.35. We can visualize estimated distributions of *Q*_*U*_ and see in Figure 4 that distributions of $C_{Eye,model}$ and $N_{Eye,model}$ scores were well-separated with wider distributions and intersected at *Q*_*U*_ = 0.53, while the estimated distribution of *Q*_*U*_ of $N_{Eye,model}$ had a more narrow distribution with relatively symmetric decreasing tails centered at 0.47.

### 2.2. Scoring Data With Multimodal Sources

In cases when the noise source cannot be captured directly from the same recording modality and require multiple recording modalities (referred to as “multimodal method”), it may not be possible to directly compare distributions of quantitative features of both the signal of interest and noise. For example, in our application scoring EEG with motion artifacts, the value of entropy of an EEG channel may not be directly comparable to the entropy of acceleration in the X-axis from an IMU. Therefore, we must compare clean signals and raw signals with noise present to formulate models to identify differences.

#### 2.2.1. Scoring Method for Data With Multimodal Sources

This problem can be formulated as a two-class classification machine learning problem. In one class we have clean data (C) and in the second class we have raw recorded data with noise present (R). For classification these two data are assigned numeric labels, {C:1, R:0}. The deep learning classifier can then find the difference between the two data sets which here is the presence of the noise. Once trained, new data can be classified with a probabilistic prediction using Softmax functions in the last layer. The closer the probability is to zero, the more similar the signal is to the noise source, while probabilities closer to one would represent predicted signals without noise.

The deep learning model would traditionally select the class with the highest probabilities as the prediction. To score the data from the deep learning models, we used the prediction probabilities directly (Equation 8). Here, $P_{C}$ is the predicted probability of input data being part of the clean data class and $P_{R}$ is the predicted probability of input data being part of the raw data class with noise present. Then the scoring function for our multimodal method is defined as

(8) $$\begin{array}{l} Q_{M}=\{\begin{array}{l} \frac{1}{2}+\frac{P_{C}-P_{R}}{2},\text{if }P_{C}\geq P_{R}\\ \frac{1}{2}-\frac{P_{R}-P_{C}}{2},\text{if }P_{C}<P_{R} \end{array} \end{array}$$

The motivation of this definition is such that if $P_{C}=1,P_{R}=0$ then *Q*_*M*_ = 1, if $P_{C}=0,P_{R}=1$ then *Q*_*M*_ = 0, and if $P_{C}=0.5,P_{R}=0.5$ then *Q*_*M*_ = 0.5. Further, when differences between the probabilities are larger, *Q*_*M*_ should be made higher when $P_{C}>>P_{R}$ and lower when $P_{C}<<P_{R}$. This is desired since larger differences between $P_{C}$ and $P_{R}$ would imply that the predictions of the model are more confident and therefore scores should be adjusted accordingly.

#### 2.2.2. Data With Multimodal Sources

To generate trained models and subsequently validate the multimodal approach, we focused on EEG with noise from motion since motion cannot be recorded directly by electrodes, as EEG and EOG are.

We again used the data set obtained from the University of Houston that contained EEG recordings (sampled at 100 Hz) as well as motion capture from eleven subjects walking on a treadmill for 6 min (section 2.1.2) (Kilicarslan and Vidal, 2019). EEG was recorded from the same electrodes and configuration referred to above with data from all eleven subjects available. Further, the experimental protocol had subjects walk on a treadmill at one, two, three, and four miles-per-hour.

Another noise removal algorithm was used to remove motion artifacts (Kilicarslan and Vidal, 2019). This algorithm also used an *H*^∞^ filter formulation with Voltera series and time-varying weight assumption. Unlike EOG data which was directly measured from the same modality, the reference signal used to identify the motion artifacts in EEG signals were 3-axis acceleration values, after gravity compensation, using the quaternion of IMUs. For more detailed derivation of the *H*^∞^ filtering formulation and algorithm targeting noise from motion used in this study, the following reference can be reviewed (Kilicarslan and Vidal, 2019).

We further supplemented this data with EEG data from another study. EEG was recorded from 20 subjects while walking around an art exhibit (Kontson et al., 2015; Cruz-Garza et al., 2017). These EEG were recorded with a 20 electrodes labeled in accordance with the extended 10-20 system as well as two electrodes for EOG, placed below the right eye and on the right temple. Each trial began with a baseline wall stair of approximately 1-min. Afterwards, subjects walked around an art exhibit for at least 7 min.

To obtain a robust model that scored EEG quality based on the presence of motion artifacts we combined data from both these sources to obtain a $C_{Motion}$ that represented EEG data from recordings where motion was removed through an artifact removal algorithm and recordings where motion was known to not be present. Similarly, we combined data from both sources to obtain a $R_{Motion}$ that represented EEG data from recordings where motion was present under different circumstances, in both controlled environments with different walking speeds, and in an uncontrolled setting where subjects walked through an art exhibit. Since data from our second source (Kontson et al., 2015) only had 20 EEG channels available, we used the same 20 channels from our first data source (Kilicarslan and Vidal, 2019). Each recording from both sources were segmented into 30-s epochs for our multimodal method. Combining these we obtained $C_{Motion}$ with *n* = 568 (12 30-s segments from 6-min ×4 walking speeds ×11 subjects + 2 30-s segments from 1-min of baseline ×20 subjects = 568), and $R_{Motion}$ with *n* = 808 (12 30-s segments from 6-min ×4 walking speeds ×11 subjects + 14 30-s segments from 7-min of walking ×20 subjects = 808), where each sample was of size 3, 000 ×20 (30-s segments sampled at 100 Hz across 20 channels), for our method scoring data with multimodal source.

#### 2.2.3. Model Generation for Data With Multimodal Sources: EEG With Motion Artifacts

To obtain scoring models for this data we separated 90% of the data randomly to generate the models and reserved 10% of the data to test and validate the results. We present 10-fold cross-validated values from ten generated models from different balanced samplings of 90% of the data, denoted with the subscript “*model,”* that resulted in $C_{Motion,model}$ and $R_{Motion,model}$, each with *n* = 512. We report the mean results as well as the mean standard deviation across samples for the 10-fold cross-validation. For this application we used deep learning models used in previous research that used multi-channel EEGs as input and output class predictions (Schirrmeister et al., 2017).

We show in Table 3 predicted probabilities and associated scores (Equation 8) using model training data from our deep learning-based scoring for our multimodal scoring method.

**Table 3:** Mean predicted probabilities ± standard deviation and corresponding mean scores, ${\bar{Q}}_{M}$, of multimodal method across model generation cross-validation.

**Data**	**Probability of $C_{Motion}$**	**Probability of $R_{Motion}$**	**${\bar{Q}}_{M}$**
$C_{Motion,model}$	0.79 ± 0.13	0.18 ± 0.12	0.81 ± 0.13
$R_{Motion,model}$	0.28 ± 0.26	0.67 ± 0.26	0.30 ± 0.26

**Figure 5:**
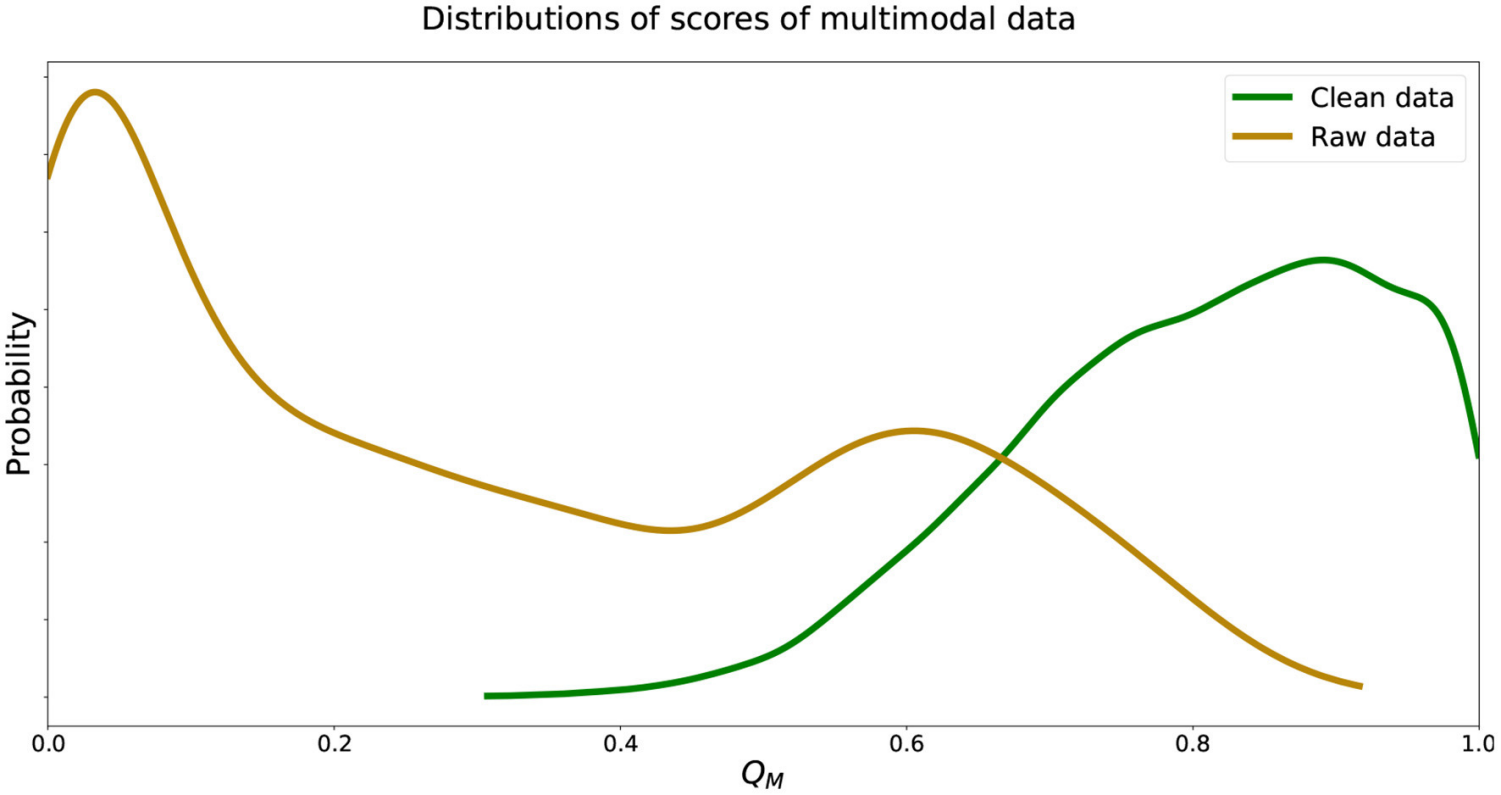
Estimated distributions of *Q*_*M*_ for $C_{Motion,model}$ and $R_{Motion,model}$. Distributions of *Q*_*M*_ of $C_{Motion,model}$ and $R_{Motion,model}$ intersect at *Q*_*M*_ = 0.62.

We see that the recorded data was in fact scored well below 0.5 while data without walking artifacts were scored well above 0.5, instilling confidence in the trained deep learning models. We visualize estimated distributions of *Q*_*M*_ and see in Figure 5 that the distribution of $C_{Motion,model}$ scores was negatively skewed, the distribution $R_{Motion,model}$ scores was positively skewed, and both distributions intersected at *Q*_*M*_ = 0.62.

### 2.3. Evaluating Artifact Removal Algorithms: Removing Ocular Artifacts From EEG

Using scoring methods introduced in this study, we can evaluate the efficacy of artifact removal algorithms that target a specific type of noise. The most common type of noise that artifact removal algorithms target in EEG recordings is from ocular activity. Therefore, we use our unimodal method (section 2.1) to evaluate these types of algorithms. A MATLAB software toolbox that is commonly used in EEG processing is EEGLAB. We used two artifact removal algorithms with different approaches that aim to remove ocular artifacts. Though different, both methods rely on independent component analysis (ICA) which is the most common method used for removing ocular artifacts (Jiang et al., 2019).

Using EEGLAB (version 14.1.1) and available plug-ins, we applied the independent component artifact classification multiple artifact rejection algorithm (MARA) to $R_{Eye}$ (Winkler et al., 2011, 2014). This method uses trained classifiers to identify components from ICA that are artifactual for rejection. The FastICA method was used for obtaining ICA components and components identified by MARA were automatically rejected (Hyvarinen, 1999).

We also applied automatic artifact removal (AAR) (version 1.3) targeting EOG removal using blind source separation (BSS) to $R_{Eye}$ (Jung et al., 2000a,b). AAR with BSS was applied with all defaults, using the SOBI algorithm in MATLAB which has been shown to be effective for BSS (Belouchrani et al., 1997; Sahonero-Alvarez and Calderon, 2017). To automatically apply AAR with BSS to EOG, the method identifies ICA components that represent EOG noise by removing components with the lowest mean fractal dimension values (Gomez-Herrero et al., 2006).

To evaluate and compare the effectiveness of these artifact removal algorithms we obtained the average score of ${\bar{Q}}_{U}\left(R_{Eye}\right)$ and compared the average scores of the data after being processed with MARA, ${\bar{Q}}_{U}\left(C_{Eye-MARA}\right)$, and ARR, ${\bar{Q}}_{U}\left(C_{Eye-AAR}\right)$.

## 3. Results

We show mean results as well as the mean standard deviation across samples for our unimodal and multimodal scoring methods for the data sets specified. For both methods, we report the 10-fold cross-validated values from 10 generated models with the remaining 10% unseen data, denoted with the subscript “*valid.”* For the unimodal method, we scored $C_{Eye,valid}$ and $R_{Eye,valid}$, both with *n* = 278, and $N_{Eye,valid}$, with *n* = 19. For the multimodal method, we scored $C_{Motion,valid}$ and $R_{Motion,valid}$, each with *n* = 56. These validation results are presented below in section 3.1 (unimodal method) and section 3.2 (multimodal method). We also validated both methods using an independent public data set from a study analyzing the differences in neural activity between motor imagery, mental arithmetic, and other artifact generating tasks (Shin et al., 2017). Each of the 29 subjects included in this study performed each of the motor imagery and mental arithmetic tasks three times and all other tasks once. These data were recorded using thirty EEG electrodes according to the 10-5 system. Different subset of the tasks were evaluated for each scoring method given data availability. Details of the analysis and data are presented in Data Availability Statement. Section 3.3 shows results using all data and validation data.

### 3.1. Scoring Data With Unimodal Source: EEG With Ocular Artifacts

For our unimodal method, in addition to the scoring models, we also formulated a criterion to quantify features that were effective for scoring EEG. Results show that the *E*_*total*_ measure and δ performance metric meaningfully represented a feature's ability to score EEG in a unimodal data source setting. We determined that features should be used only if they have a *E*_*total, f*_ ≤ 0.35. From analyses shown in Figure 2 and Table 2, we see that features with *E*_*total*_, ≤ 0.35 performed better than those with higher *E*_*total*_. Incorporating features with 0.35 < *E*_*total, f*_ ≤ 0.50 along with features with lower *E*_*total, f*_ decreased performance.

Applying the unimodal method to data not used for model development we scored the data (Equation 2) and present cross-validation results using features that met the inclusion criteria determined for features, *E*_*total, f*_ ≤ 0.35 (Table 4).

**Table 4:** Mean scores $\bar{Q_{U}}$ ± standard deviation and δ values of unimodal method results across cross-validation using features *E*_*total, f*_ ≤ 0.35.

**Data**	**${\bar{Q}}_{U}$**
	**(*E*_*total, f*_ ≤ 0.35)**
$C_{Eye,valid}$	0.73 ± 0.11
$R_{Eye,valid}$	0.50 ± 0.14
$N_{Eye,valid}$	0.23 ± 0.14
δ	0.04

We see that the models developed performed well, obtaining δ = 0.04 from ideal mean score characteristics. The ${\bar{Q}}_{U}$ and δ found using the unseen validation data were similar to those from data used generating these models. Further, estimated distributions of *Q*_*U*_ of validation data followed closely those shown in Figure 4 with distributions of *Q*_*U*_ of $C_{Eye,valid}$ and $N_{Eye,valid}$ intersecting at 0.52.

Further, we validated our unimodal method on an independent open source data (Shin et al., 2017). The data was annotated such that EEG data was available under five different conditions: (1) subjects instructed to blink at one second intervals for 20 s (Blinking), (2) subjects instructed to look at a moving dot that moved around at four locations on the screen at 2 s intervals, repeated 5 times (Eye movements), (3) subjects instructed to move their heads in four directions at 2 s intervals, repeated 5 times (Head movements), (4) subjects performing motor imagery tasks (Motor imagery), (5) subjects performing mental arithmetic tasks (Mental arithmetic), and (6) subjects instructed to gaze at cross-hairs before trials during tasks (Gazing). These five conditions presented tasks with different levels of expected noise which could be used to evaluate the effectiveness of our unimodal model on a second, completely independent dataset. We scored all data available of each type which are shown in Table 5.

**Table 5:** Comparison of mean scores ${\bar{Q}}_{U}$ ± standard deviation of unimodal method across cross-validation for all data from independent validation data.

**Task**	**${\bar{Q}}_{U}$**
Blinking	0.23 ± 0.16
Eye movements	0.34 ± 0.12
Head movements	0.39 ± 0.13
Motor imagery	0.40 ± 0.14
Mental arithmetic	0.40 ± 0.14
Gazing	0.52 ± 0.17

We see that as expected, the blinking and eye movement data fall in the 0 to 0.5 range of our metric, as our $N_{Eye}$ data, since they generally represents noise. The remaining data fall near the middle of the 0.25 to 0.75 range of our metric, as our $R_{Eye,valid}$, since they have both EEG and noise from ocular artifacts present. We also see that the moments when subjects were instructed to gaze, for the purposes of reducing eye movements, our unimodal method produced higher scores.

### 3.2. Scoring Data With Multimodal Sources: EEG With Motion Artifacts

We next show results of our multimodal data scoring method. We show the cross-validated predicted probabilities for each data type as well as the quality score (Equation 8) using our deep learning-based scoring method on unseen data (Table 6).

**Table 6:** Mean predicted probabilities ± standard deviation and corresponding mean scores, ${\bar{Q}}_{M}$, of multimodal method results across cross-validation.

**Data**	**Probability of $C_{Motion}$**	**Probability of $R_{Motion}$**	**${\bar{Q}}_{M}$**
$C_{Motion,valid}$	0.77 ± 0.13	0.19 ± 0.12	0.79 ± 0.13
$R_{Motion,valid}$	0.28 ± 0.27	0.67 ± 0.28	0.31 ± 0.28

We see that the predicted probabilities and ${\bar{Q}}_{M}$ from unseen data were similar to those from the model generation data. The model was able to score recorded data with noise well below 0.5 while also scoring clean data well above 0.5. Estimated distributions of *Q*_*M*_ of unseen validation data followed closely those shown in Figure 5 with distributions of *Q*_*M*_ of $C_{Motion,valid}$ and $R_{Motion,valid}$ intersecting at 0.57. We note that mean cross-validated standard deviations of ${\bar{Q}}_{M}\left(R_{Motion}\right)$ data were twice as large as ${\bar{Q}}_{M}\left(C_{Motion}\right)$ data, which we discuss in section 4.

Further, we also validated our multimodal method on an independent open source data (Shin et al., 2017). Though the data was annotated such that EEG data was available under five different conditions, here we score only the two task conditions: (1) subjects performing motor imagery tasks (Motor imagery), and (2) subjects performing mental arithmetic tasks (Mental arithmetic). This was done since 30 continuous seconds of data was not available under the other conditions. These two conditions present two tasks with similar levels of expected noise from motion to evaluate the effectiveness of our multimodal model on a second, completely independent dataset. We scored all data available of each type which are shown in Table 7.

**Table 7:** Comparison of mean scores ${\bar{Q}}_{M}$ ± standard deviation of multimodal method across cross-validation for task data from independent validation data.

**Task**	**${\bar{Q}}_{M}$**
Motor imagery	0.93 ± 0.14
Mental arithmetic	0.94 ± 0.12

We see that as expected, the data from tasks done in this experiment, which were conducted while seated, obtained high scores from our metric, close to 1, similar to our $C_{Motion}$ data, since they generally did not have noise from motion.

### 3.3. Evaluating Algorithms Removing Ocular Artifacts From EEG

As an extension and further application of our unimodal scoring method we score $R_{Eye}$ data with artifacts removed by two different methods, MARA and AAR (section 2.3). To compare their effectiveness we score all $R_{Eye}$ data (section 2.1.2), with *n* = 2, 784, and validation $R_{Eye,valid}$ data (section 3.1), with *n* = 278 (Table 8).

**Table 8:** Comparison of mean scores ${\bar{Q}}_{U}$ ± standard deviation of unimodal method across cross-validation of all data and validation data processed by MARA and AAR methods.

**Data**	**${\bar{Q}}_{U}$**
$C_{Eye-MARA}$	0.72 ± 0.12
$C_{Eye-AAR}$	0.60 ± 0.11
$R_{Eye}$	0.50 ± 0.14
$C_{Eye,valid-MARA}$	0.72 ± 0.10
$C_{Eye,valid-AAR}$	0.61 ± 0.10
$R_{Eye,valid}$	0.50 ± 0.14

We see that for both sets of data, ${\bar{Q}}_{U}\left(C_{Eye-MARA}\right)>{\bar{Q}}_{U}\left(C_{Eye-AAR}\right)$. That is, data processed with MARA, $C_{Eye-MARA}$, was scored higher than data processed with AAR, $C_{Eye-AAR}$. This means that MARA may have been more effective than AAR at removing ocular artifacts from EEG. Further, as hypothesized, data processed by both artifact removal algorithms resulted in data with mean scores higher than ${\bar{Q}}_{U}\left(R_{Eye}\right)$.

## 4. Discussion

We present in this work two novel methods to score electrophysiological data signal quality. In the first method, we quantify signal quality when the noise source (e.g., ocular artifacts) could be recorded from the same modality (i.e., electrodes on the head) as the signal of interest. In such cases, the same quantitative features can generally be computed on both the signal of interest (EEG) and noise source (EOG), and compared directly to each other. For other sources of artifacts such as motion, supplemental physiological measurement tools would be needed to measure artifact signals directly. In these cases, it is not usually possible to compare quantitative features computed on both the signal of interest and noise source directly. Therefore, in the second method, the noise source signal is not needed; rather only data with and without the noise are required.

With both methods, we generate models to score signal quality of EEG with either ocular or motion artifacts. Though high density EEG headsets with electrodes to measure EOG are commonly used in research settings, low density headsets are becoming more common and have been shown to be effective for many applications (Justesen et al., 2019). Even when high density EEG headsets are used, other artifact sources, like motion, are not generally directly captured. These data-driven models, once generated, can be used to evaluate the quality of EEG and potential presence of noise from artifacts of other data without the need to directly record noise sources.

In general, our unimodal method was able to generalize to data not used in generating the scoring models. Results in Table 4 and score distributions matched values found from generating these models in Table 2 and shown in Figure 4. For our multimodal method, we were able to score data from two different data sets well. Combined data that had artifacts removed and baseline data when subjects were known to be still were both scored as generally clean data. Both in the model generation and validation of this method, we found that the cross-validated standard deviations of recorded data scores, ${\bar{Q}}_{M}\left(R_{Motion}\right)$, were twice as large as standard deviations of clean data scores, ${\bar{Q}}_{M}\left(C_{Motion}\right)$ (Tables 3, 6). We also saw a dual-mode distribution of $Q_{M}\left(R_{Motion}\right)$ scores, with peaks around 0.05 and 0.65 (Figure 5). The large standard deviation and dual-mode distribution may be due to variation in the speed and direction of subject walking within data sources used. Some subjects walked at various speeds (1–4 mph) while others walked through an art exhibit at their own pace, even potentially with stops and turns to view art pieces (Kontson et al., 2015; Kilicarslan and Vidal, 2019). Future work could separate out these data of different walking types to further validate the accuracy and effectiveness of the scoring model.

We also validated our unimodal and multimodal methods on an independent data set comprised of recordings from subjects that were instructed to perform several tasks, including tasks intended to generate ocular artifacts (Shin et al., 2017). Results from scoring these independent data showed that our methods appropriately scored each type of data for quality considering either noise from ocular (Table 5) or motion (Table 7) artifacts. Our scores showed that when instructed to perform tasks to generate ocular artifacts, unimodal scores were lower and when performing tasks of interest there was noise from ocular artifacts present, which was acknowledged and removed in the original study's analysis (Shin et al., 2017). Our multimodal method's effectiveness was also further supported since subjects were seated during the study and would have been expected that the data have little to no noise from motion.

Examining the application of our unimodal method to evaluate the effectiveness of artifact removal algorithms, we see that MARA was more effective than AAR in removing artifacts, obtaining higher scores for the processed recorded data. Strictly speaking, this means that MARA removed ocular artifacts more similarly to the *H*^∞^ method used to develop our models than AAR did. Further, we see that these results were consistent both across all data, including those used to generate our models, and the independent validation data (Table 8). These results present a significant advancement to evaluating artifact removal algorithms by providing quantitative measures on real EEG recordings as opposed to qualitative evaluations or using synthetic data (Islam et al., 2016).

Both methods were applied to EEG to score different recordings with different noise artifacts. However, future work may apply multiple models to the same recordings to obtain several quality scores that may be combined to assess an overall data quality score. We also note that though the unimodal method can only be applied when the noise source is available from the same modality, the multimodal method can be applied to data with unimodal source. Our multimodal approach, which is deep learning-based, requires a large amount of data to generate accurate models. If more data were available, we may attempt to apply our multimodal method to score data from a unimodal source, in this case using only $C_{Eye}$ and $R_{Eye}$ to score these data with *Q*_*M*_.

Comparing these approaches, we see that our multimodal method required only an appropriate deep learning model designed to classify the input signal. These deep learning models identify signal features important to distinguish the two input classes, clean signals and signals with noise, automatically. Our unimodal method on the other hand, required more manual selection of both the features of the signal to compute and feature importance for scoring signal quality. However, as opposed to our multimodal method, our unimodal method allows for the identification of specific quantitative features of the signal of interest that were important for scoring signal quality with respect to the targeted noise.

Both our unimodal and multimodal methods present advancements and improvements from existing methods by evaluating EEG signal quality with continuous scores. Previous methods have generally evaluated signal quality by classifying EEG into discrete quality categories (Daly et al., 2012; Hu et al., 2013; Grosselin et al., 2019). Our scoring models allow for rapid evaluation of signal quality of EEG data. Future work may expand the type of data used to generate these models, such as including signals known to have no ocular artifacts present. To further validate scores generated by these models, data could be obtained or generated with known levels of noise. Signals with more noise introduced should result in lower scores. Our analyses presented signals of each type in aggregate, averaging across samples, future analyses may investigate recordings more specifically to identify the level of noise in signals.

## Data Availability Statement

The original contributions presented in the study are publicly available. Software, scripts, and models from this study are available at our GitHub Repository: https://www.github.com/dbp-osel/EEG-quality. Three datasets were used in this study. Two datasets are publicly available and the sources are included in the article. The other data were provided directly by the University of Houston, which has published work on this data and is referenced in the article. Requests to access these datasets should be directed to Jose L. Contreras-Vidal, jlcontr2@central.uh.edu.

## Ethics Statement

For the studies involving human participants, the experimental protocol and anonymous informed consent were approved by the University of Houston's (UH) Institutional Review Board (IRB). De-identified data was provided by UH for secondary analysis in this study. The patients/participants provided their written informed consent to participate in this study.

## Author Contributions

KK and DN: conceptualization, methodology, validation, writing—reviewing and editing, and visualization. DN: software, formal analysis, investigation, and writing—original draft. KK: resources, supervision, and funding acquisition.

## Conflict of Interest

The authors declare that the research was conducted in the absence of any commercial or financial relationships that could be construed as a potential conflict of interest.
